# Association Between State Indoor Tanning Legislation and Google Search Trends Data in the United States From 2006 to 2019: Time-Series Analysis

**DOI:** 10.2196/26707

**Published:** 2021-04-09

**Authors:** Carolyn Heckman, Yong Lin, Mary Riley, Yaqun Wang, Trishnee Bhurosy, Anna Mitarotondo, Baichen Xu, Jerod Stapleton

**Affiliations:** 1 Rutgers Cancer Institute of New Jersey New Brunswick, NJ United States; 2 Medtronic Denver, CO United States; 3 University of Kentucky Lexington, KY United States

**Keywords:** adolescents, dermatology, Google Trends, indoor tanning, internet, policy, prevention, skin cancer, skin cancer prevention, tanning, trend, time series, web-based health information, young adult, youth

## Abstract

**Background:**

Exposure to ultraviolet radiation from the sun or indoor tanning is the cause of most skin cancers. Although indoor tanning has decreased in recent years, it remains most common among adolescents and young adults, whose skin is particularly vulnerable to long-term damage. US states have adopted several types of legislation to attempt to minimize indoor tanning among minors: a ban on indoor tanning among all minors, a partial minor ban by age (eg, <14 years), or the requirement of parental consent or accompaniment for tanning. Currently, only 6 US states have no indoor tanning legislation for minors.

**Objective:**

This study investigated whether internet searches (as an indicator of interest) related to indoor tanning varied across US states by the type of indoor tanning legislation, using data from Google Trends from 2006 to 2019.

**Methods:**

We conducted a time-series analysis of Google Trends data on indoor tanning from 2006 to 2019 by US state. Time-series linear regression models were generated to assess the Google Trends data over time by the type of indoor tanning legislation.

**Results:**

We found that indoor tanning search rates decreased significantly for all 50 states and the District of Columbia over time (*P*<.01). The searches peaked in 2012 when indoor tanning received marked attention (eg, indoor tanning was banned for all minors by the first state—California). The reduction in search rates was more marked for states with a complete ban among minors compared to those with less restrictive types of legislation.

**Conclusions:**

Our findings are consistent with those of other studies on the association between indoor tanning regulations and attitudinal and behavioral trends related to indoor tanning. The main limitation of the study is that raw search data were not available for more precise analysis. With changes in interest and norms, indoor tanning and skin cancer risk among young people may change. Future studies should continue to determine the impact of such public health policies in order to inform policy efforts and minimize risks to public health.

## Introduction

Ultraviolet radiation from the sun or indoor tanning is the cause of most skin cancers [1]. Although indoor tanning has decreased in recent years, it remains most common among adolescents and young adults [2,3], whose skin is particularly vulnerable to long-term damage [1]. US states have adopted several types of legislation to minimize indoor tanning among minors: a ban on indoor tanning among all minors, a partial minor ban by age (eg, <14 years), or the requirement of parental consent or accompaniment during indoor tanning. Currently, only 6 US states (Alaska, Colorado, Iowa, Montana, New Mexico, and South Dakota) have no indoor tanning legislation for minors [4]. The increase in indoor tanning restrictions may explain reductions in the number of indoor tanning providers, consumer spending on indoor tanning [5], and past-year indoor tanning among girls (24.1% in 2009 and 9.5% in 2015) and boys (5.7% in 2009 and 3.3% in 2015) attending high school and young adults aged 18-34 years (14% in 2007 and 4% in 2018) in the United States in recent years [3,6]. More stringent regulations have been associated with greater reductions in indoor tanning behavior and have been estimated to have a greater impact on melanoma incidence, mortality, and cost [7-10].

Internet search trends indicate public interest in a topic and are associated with actual health-related events such as influenza and COVID-19 outbreaks [11,12], medication use [13,14], melanoma mortality rates [15], and tobacco- and alcohol-related policy changes [16,17]. This study investigated whether internet searches (as an indicator of interest) related to indoor tanning varied across US states by the type of indoor tanning legislation, using free, publicly available data from Google Trends from 2006 to 2019. We hypothesized that the reduction in search rates over time would be associated with stricter indoor tanning regulations (eg, a ban on indoor tanning among all minors).

## Methods

Data were downloaded from Google Trends [18]; these data reflect how many searches have been conducted on a specified topic relative to the total number of searches on Google within the selected time frame and geographic location. Search volume indices range from 0 (no searches) to 100 (peak number of searches). We selected the topic “indoor tanning,” which includes related search terms (eg, “tanning bed”). The indoor tanning time series consists of search volume indices from January 2006 to October 2019 for each state, along with the District of Columbia, and the United States as a whole. Google Trends data were available for 2004 and 2005, but state data were not sufficient for analysis.

In order to study longitudinal trends, seasonal effects were first excluded from the time series, since indoor tanning is most popular during spring in many parts of the United States [19]. We fitted 2 linear models for each state to evaluate the Google Trends data on indoor tanning and their association with the legislation type (a ban of all minors [n=22], a partial ban [n=10], requirement of parental consent [n=13], and no legislation [n=6] as of October 2019) as documented by the National Conference of State Legislatures [4]. Model 1 is a change-point model with the date of legislation enactment as the change point and as an outlier, since we observed an additional peak for some states on the date of legislation enactment. The first legislation was enacted in Wisconsin in 1991 (a partial ban), and the latest legislation included in the analyses were those enacted in Maine and Maryland (complete bans) in September and October of 2019, respectively. Model 2 is a model without any change points. To account for the correlations among adjacent time points in both models, an autoregressive moving average error structure was used. The fitted slopes (change rates) for all states in both models were calculated. The association between legislation type and the fitted slopes was assessed using a heterogenous variance model owing to unequal variations in slopes among legislation types. For Model 1, the difference in slope before and after the date of legislation enactment was first evaluated to determine whether Model 2 was sufficient for comparisons among legislation types. For multiple comparisons, *P* values were adjusted on the basis of the Tukey method for multiplicity adjustment. A *P* value less than .05 was considered significant. Statistical analyses were conducted using R (version 4.0.1, The R Foundation) [20] and SAS (version 9.4, The SAS Institute) [21].

## Results

**Figure 1:**
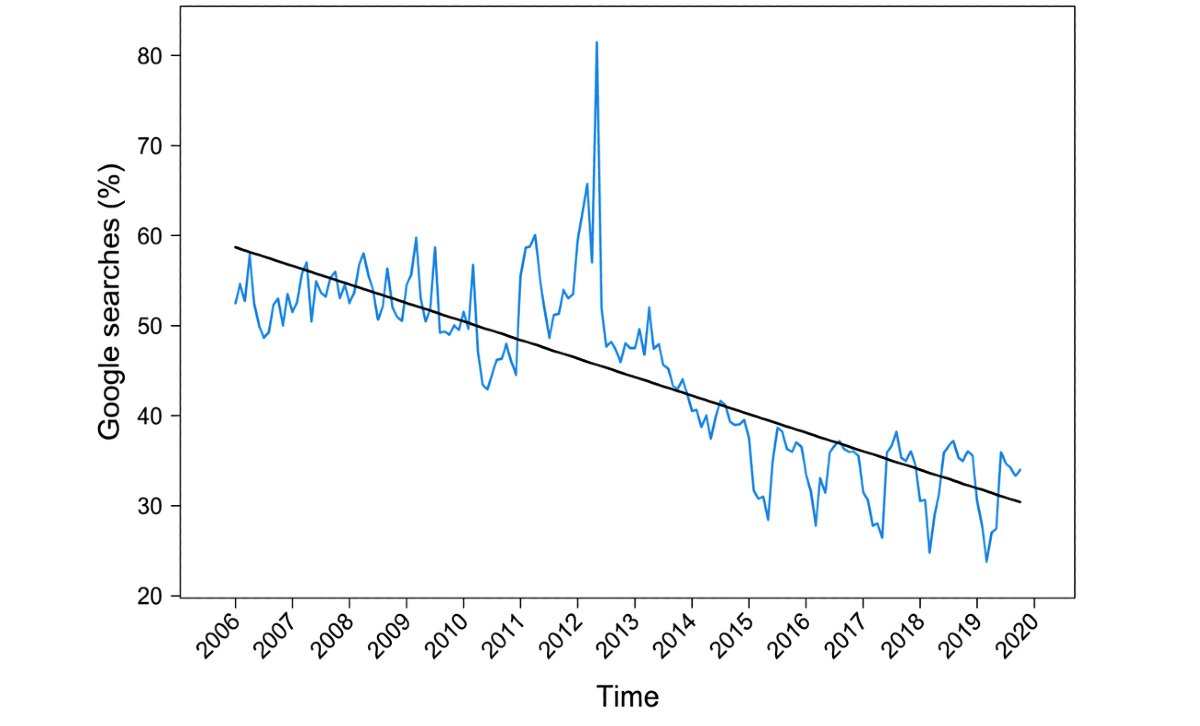
Google Trends data for the United States with fitted regression lines. Blue line: time series data after excluding seasonal effects, black line: Model 2 without change points.

Figure 1 shows the trend in Google searches related to indoor tanning for the United States overall with the fitted regression line for the no-change-point model. The slope (change rate for tanning search trends) decreased over time. When individual states (Multimedia Appendix 1) were grouped by legislation type, the decreasing slope rates differed. These decreasing rates were greater for states that imposed a ban on all minors than for states with other types of legislation. We calculated *P* values to compare slopes before and after the date of legislation enactment, based on change-point Model 1, for states that imposed a minor ban. Before multiplicity adjustment, *P* values were significant for only the District of Columbia (2015), Delaware (2015), and Nebraska (2014) (*P*=.02-.04). After multiplicity adjustment, all these *P* values were not significant (*P≥*.44). In addition, the slope differences before and after the enactment of the legislation by legislation type were not significant (*P*=.84). Hence, we only compared the legislation types in accordance with Model 2.

As indicated in Model 2, the legislation type was significantly associated with a reduction in search rates (slopes) (*P*=.01). Table 1 shows the results of pairwise comparisons in the reduction in search rates by legislation type. We found that the reduction in the indoor tanning search trend was more marked for states that imposed a ban on all minors (0.6% per month smaller search volume) than for those that imposed a partial ban (*P*=.009, adjusted *P*=.04). Furthermore, this rate reduction was more marked for states that imposed a ban on all minors (0.5% per month smaller search volume) than for those that required parental consent (*P*=.005, adjusted *P*=.02). Moreover, this rate reduction was more marked for states that imposed a ban on all minors (0.2% per month smaller search volume) than for those with no legislation; these values were borderline significant before adjustment but did not significantly differ after multiplicity adjustment (*P*=.08, adjusted *P*=.28). The rate reduction was more marked for states with no legislation than for those requiring parental consent and those that imposed a partial ban, but these values did not significantly differ after multiplicity adjustment (adjusted *P*≥.22). Finally, rate reductions between states requiring parental consent and those that imposed a partial ban did not significantly differ (*P*=.22, adjusted *P*=.98).

**Table 1:** Pairwise comparisons of Google Trends search data by legislation type.

Comparisons	Rate difference (SE)	*P* value^a^	Adjusted *P* value^b^
States that banned all minors vs states that required parental consent	–0.60 (0.22)	*.009*	*.04*
States that banned all minors vs states that imposed a partial ban	–0.51 (0.17)	*.005*	*.02*
States that banned all minors vs states with no legislation	–0.27 (0.13)	.08	.28
States with no legislation vs states that required parental consent	–0.34 (0.20)	.07	.27
States with no legislation vs states that imposed a partial ban	–0.24 (0.14)	.06	.22
States that required parental consent vs states that imposed a partial ban	0.09 (0.22)	.70	.98

^a^Significant *P* values are italicized.

^b^Values are based on Tukey-Kramer adjustment.

## Discussion

### Principal Findings

Studies have previously analyzed Google Trends data related to tanning, skin protection, skin cancer, and other health-related issues, along with tanning trends by season, geographic location, and population demographics of US states [15,19,22-24]. However, to our knowledge, no previous studies have explored an association between Google search rates and indoor tanning–related legislation. This study shows that indoor tanning search rates decreased significantly for all 50 US states and the District of Columbia over time. We observed a peak in 2012 when indoor tanning received increased media attention. For example, in 2012, along with the release of the final season of the television show Jersey Shore (catchphrase: “Gym, Tan, Laundry”), Patricia Krentcil from New Jersey was accused of bringing her fair-skinned, red-headed, 5-year-old daughter to tanning salons with her, and indoor tanning was banned for all minors by the first state—California.

The reduction in the Google search rate was more marked for states that imposed bans among all minors than for those with a less restrictive legislation. Considering the limitations of Google Trends data and the wide variation in the timing of legislation across US states, there are several potential explanations for these findings. For example, restrictive regulations may influence interest in tanning, as evidenced by internet search trends, or decreased interest in tanning may facilitate the enactment of more restrictive policies. These associations may also be better accounted for by other unmeasured factors (eg, increasing outdoor temperatures over time). It is not surprising that we observed no significant difference in search trends for states that imposed partial bans and those that require parental consent or accompaniment, since both types of policies are partial restrictions. However, it is difficult to explain the lack of a significant difference in the trends for states with no legislation and those with other types of legislation. Perhaps search trends for states with no legislation are more likely to be similar to the nationwide media trends if state and local media attention is limited.

### Strengths and Limitations

The strengths of this study include its longitudinal analysis of a nationwide data set based on millions of Google searches. A key limitation of the study is that raw search data were not available for more precise analyses. The data are anonymized; hence, we are unaware of the demographics or other characteristics of the searchers, including (for example) what proportion of searchers are youth or adults or are for, against, or neutral toward indoor tanning. The data are limited to the 90% of people in the United States who use the internet [25] and the 88.1% of internet searches conducted on Google [26], which tends to be more representative of people aged under 45 years, compared to other search engines such as Bing or Yahoo [27]. Arora et al [28] have previously reviewed the potential opportunities and limitations of Google Trends data for use in health and health policy research.

### Conclusions

In the context of other relevant data, Google Trends data may provide novel insights into health- and health policy–related trends. Longitudinal Google search trends are associated with the type of indoor tanning legislation. As interest in tanning and norms change, indoor tanning and the skin cancer risk among young people may also change [3]. Future studies should continue to investigate the impact of such public health policies to inform policy efforts and minimize the public health risk.

## Appendix

Multimedia Appendix 1 Google search trends with fitted regression lines (Black line: simple linear regression; Red line: Change-point linear regression; Blue line: Time series data after removing seasonal effect).
