# Correction: Association Between State Indoor Tanning Legislation and Google Search Trends Data in the United States From 2006 to 2019: Time-Series Analysis

**DOI:** 10.2196/29516

**Published:** 2021-04-14

**Authors:** Carolyn Heckman, Yong Lin, Mary Riley, Yaqun Wang, Trishnee Bhurosy, Anna Mitarotondo, Baichen Xu, Jerod Stapleton

**Affiliations:** 1 Rutgers Cancer Institute of New Jersey New Brunswick, NJ United States; 2 Medtronic Denver, CO United States; 3 University of Kentucky Lexington, KY United States

In “Association Between State Indoor Tanning Legislation and Google Search Trends Data in the United States From 2006 to 2019: Time-Series Analysis” (JMIR Dermatol 2021;4(1):e26707) the authors noted two errors.

Due to a system error, the name of one author, Trishnee Bhurosy, was replaced with the name of another author on the paper, Jerod Stapleton. In the originally published paper, the order of authors was listed as follows:

Carolyn Heckman; Yong Lin; Mary Riley; Yaqun Wang; Jerod Stapleton; Anna Mitarotondo; Baichen Xu; Jerod Stapleton

This has been corrected to:

Carolyn Heckman; Yong Lin; Mary Riley; Yaqun Wang; Trishnee Bhurosy; Anna Mitarotondo; Baichen Xu; Jerod Stapleton

In the originally published paper, the ORCID of author Trishnee Bhurosy was incorrectly published as follows:

Trishnee Bhurosy: 0000-0002-8501-1483

This has been corrected to:

Trishnee Bhurosy: 0000-0003-2603-2839

The correction will appear in the online version of the paper on the JMIR Publications website on April 14, 2021, together with the publication of this correction notice. Because this was made after submission to PubMed, PubMed Central, and other full-text repositories, the corrected article has also been resubmitted to those repositories.

