# Supplementary material for: Association Between State Indoor Tanning Legislation and Google Search Trends Data in the United States From 2006 to 2019: Time-Series Analysis
Source: JMIR Dermatol. 2021 Apr 9;4(1):e26707. doi: 10.2196/26707 (PMC10501525; doi:10.2196/26707)

eFigure 1. Google search trends with fitted regression lines (Black line: simple linear regression; Red line: Change-point linear regression; Blue line: Time series data after removing seasonal effect).


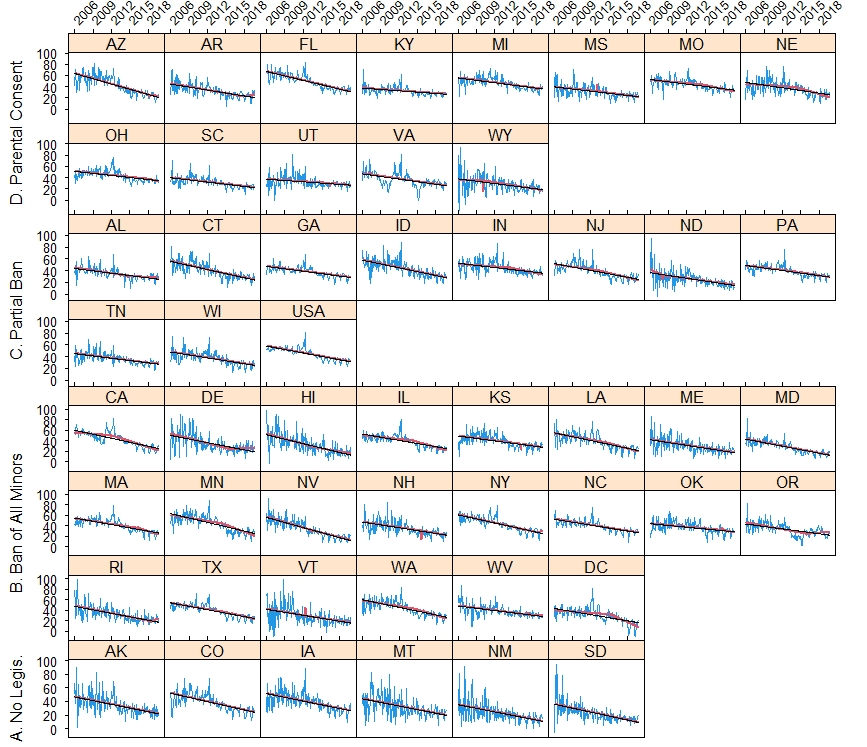

Supplement: Multimedia Appendix 1 [file derma_v4i1e26707_app1.docx]
